# Biomarkers of macular neovascularisation activity using optical coherence tomography angiography in treated stable neovascular age related macular degeneration

**DOI:** 10.1186/s12886-022-02749-5

**Published:** 2023-02-14

**Authors:** Daren Hanumunthadu, Azahir Saleh, Daniela Florea, Konstantinos Balaskas, Pearse A Keane, Tariq Aslam, Praveen J. Patel

**Affiliations:** 1 NIHR Biomedical Research Centre at Moorfields Eye Hospital NHS Foundation Trust and UCL Institute of Ophthalmology, London, UK; 2 Royal Free London NHS Foundation Trust, London, UK; 3 Manchester Royal Eye Hospital, NHS Central Manchester University Hospitals and University of Manchester, Manchester, UK

**Keywords:** Age-related macular degeneration

## Abstract

**Background:**

The aim of this study was to describe features of disease activity in patients with treated stable macular neovascularisation (MNV) in neovascular age related macular degeneration (nAMD) using optical coherence tomography angiography (OCTA).

**Methods:**

Thirty-two eyes of 32 patients with nAMD were included in this prospective, observational study. These patients were undergoing treatment with aflibercept on a treat-and-extend regimen attending an extension to a 12-week treatment interval.

**Results:**

All subjects had no macular haemorrhage and no structural OCT markers of active MNV activity at the index 12-week treatment extension visit. 31/32 OCTA images were gradeable without significant imaging artefact. The mean MNV size was 3.6mm^2^ ± 4.6mm^2^ and 27 (87.1%) had detectable MNV blood flow. 29/31 (93.5%) subjects had MNV with mature phenotypes including 10 non-specific, 10 tangle and 3 deadtree phenotypes. MNV halo and MNV central feeder vessel were noted in 18 (58.1%) and 19 (61.3%) of subjects respectively; only 1 (3.2%) subject was noted to have a MNV capillary fringe.

**Conclusions:**

MNV blood flow is still detectable using OCTA in the majority of subjects in this study with treated stable MNV. OCTA features associated included MNV mature phenotype, MNV feeder vessel, MNV halo and absence of capillary fringe.

**Supplementary Information:**

The online version contains supplementary material available at 10.1186/s12886-022-02749-5.

## Backround

Treatment of neovascular age-related macular degeneration (nAMD) with intravitreal anti-vascular endothelial growth factor (anti-VEGF) agents is now well established as current practice. [1] Treatment decisions are guided by clinical assessment, most commonly by anatomical features of macular neovascularization (MNV) disease activity using structural optical coherence tomography (OCT) imaging. In the United Kingdom, patients are often treated in the so-called “treat -and-extend” regimen where the interval between successive anti-VEGF injection treatment is increased if MNV disease activity appears to be stable. [2] Although this has led to treatment outcomes more comparable to clinical trial experience, the retinal imaging biomarkers that suggest disease stability deserve further evaluation in order to understand when treatment might safely be stopped or paused whilst optimizing visual acuity outcomes. In patients with treated stable nAMD in particular, evaluation of the likelihood of reactivation of MNV activity using new biomarkers of disease activity, would help to define the need for treatment and earlier clinical assessment. [3]

Optical coherence tomography angiography (OCTA) is a non-invasive imaging technique already available clinically to allow flow detection within retinal microvasculature without conventional dye-based angiography. [4, 5] OCTA enables concurrent visualization of blood flow by repeated sequential segmental OCT cross-sectional imaging at the same anatomical location. [6] It can be used to identify MNV in patients with nAMD but its use in the evaluation of disease activity in treated-patients requires further assessment. [7–9] OCTA disease activity in nAMD has, for example, been correlated with quantitative features such as MNV lesion size and the presence of blood flow on OCTA imaging. [10, 11] There has been much interest in qualitative features of MNV and the description of specific anatomical microvascular phenotypes, such as seafan, medusa or glomerular morphologies, have been described. [12]

Analysis of MNV microstructure using OCTA techniques has suggested that a reduction in peripheral vascular branching and anastomoses with concurrent development of a central feeder vessel were both associated with the development of disease stability. [13] A previous study has indicated that these peripheral anastomotic arcade vessels, sometimes described as a “capillary fringe” were predictive of active disease requiring treatment. [14] Indeed, previous studies have described the development of MNV maturity during treatment with anti-VEGF agents. Remodeling of vascular structure with an associated reduction in vascular tree branching has been described in OCTA imaging analysis in nAMD. [15] Furthermore, this mature phenotype development has also been identified after treatment with anti-VEGF treatment despite an increase in total MNV lesion size. [16] Absence of a peripheral hyoporeflective “halo” and a denser vascular network within the MNV have been reported to be indicative of persistent disease activity. [17, 18]

Evaluation of OCTA biomarkers of nAMD disease activity could help guide treatment regimens with anti-VEGF agents and is particularly important when considering how to manage patients who have stable disease activity on treat-and-extend-regimens. New OCTA biomarkers of disease activity could support structural OCT features in helping to distinguish eyes with a high chance of needing continued or more intensive treatment from eyes where a pause in treatment may be considered. The aim of this study was to investigate OCTA biomarkers of MNV disease activity in patients with stable treated nAMD.

## Methods

This study formed part of the *Defining Disease Activity in Neovascular AMD with Optical Coherence Tomography imaging* (DANA) study. This was a single-center prospective, observational imaging study whose aim was to describe OCTA patterns of disease activity and quiescence in treated eyes with nAMD. All study participants provided written informed consent prior to commencement of the study. This study was approved by our local ethics committee and adhered to the tenets set forth in the Declaration of Helsinki.

### Study population

Patients were identified from medical retina clinics at Moorfields Eye Hospital, London, United Kingdom. Subjects were eligible if aged more than 50 years old and were receiving aflibercept treatment for nAMD with a treat-and-extend where an extension to a 12-week interval between aflibercept injections had been achieved. Subjects had no sign of intra/subretinal fluid in structural OCT imaging and no evidence of macular haemorrhage on slit lamp biomicroscopy. Subjects were required to have visual acuity (VA) or 20/200 Snellen Acuity (Early Treatment of Diabetic Retinopathy Study (ETDRS) letter score of 35). All subjects were required to understand the nature and purpose of the study and ability to undergo the required study retinal imaging. Patients with pachychoroid and polypoidal choroidal vasculopathy were excluded from this study. Other exclusion criteria included any condition that would lead to image artefact or poor OCT image quality and any condition which, in the investigators’ opinion, would prevent the subject from completion of the required procedures, schedule or study assessments.

### Study visits

Subjects were assessed at the visit after a 12-week treatment interval. Demographic data, VA and history of previous macular treatment were collected from the electronic patient record (OpenEyes, Openeyes United Kingdom). All subjects were adequately dilated with g.tropicamide 1% and g.phenylephrine 2.5% (Chauvin Pharmaceuticals, France) applied topically prior to imaging. Subjects underwent OCT imaging as per the study protocol.

### OCT imaging protocol

All subjects underwent spectral-domain OCT and OCTA imaging on Spectralis OCT (Heidelberg Engineering, Heidelberg Germany) completed by a clinical-trials certified ophthalmic technician. All OCT images were macular volume scans centered on the fovea (20 x 20) with 49 sections and 29 frames. All OCTA images were acquired in OCTA-HR10 centered on the fovea. All subsequent images were completed in follow-up mode with baseline image set as reference.

### OCT and OCTA evaluation

All OCT and OCTA images were evaluated for signs of nAMD disease activity by the Moorfields Eye Hospital Reading Centre. A detailed grading protocol was developed with definitions and reference images for all the grading fields included in the analysis (see supplementary File). A training and accreditation process was conducted as per standard operating procedure at the Moorfields Reading Centre. The grader independently assessed 15 OCTA cases on the basis of the grading protocol and recorded the corresponding grading fields. These were then discussed in detail with the Reading Centre Director to provide feedback, reinforce accurate interpretation approaches and provide advice and guidance for improvement when needed. For accreditation, the grader assessed 10 different OCTA cases and the recorded gradings were assessed for accuracy with an accreditation threshold of 95% correct gradings as per the grading protocol. The process would be repeated until the accreditation threshold is reached. The same approach was followed for OCT interpretation. Image size was determined using the inbuilt surface area measurement functionality of the proprietary software. We conducted single grading with adjudication of each case by the Reading Centre Director.

OCT images were assessed for signs of MNV disease activity including presence of macular edema (subretinal and intraretinal fluid, new haemorrhage / increase in PED height) and for other anatomical features including subretinal hyperreflective material, PED and outer retinal tubulation.

We applied the most broadly accepted nomenclature and feature definitions for OCTA interpretation by the international retinal expert community at the time of study conduct. OCTA images were analysed for the presence and size of MNV. These OCTA images of MNV were then assessed for lesion morphology, detection of MNV blood flow and putative anatomical features of disease activity (presence of hyporeflective halo, peripheral capillary fringe and central feeder vessel).

The review process for OCTA in the DANA study, which has been broadly adopted as the preferred approach also in clinical practice, was dynamic and pro-active, not reliant on specific pre-segmented slabs. The guiding principles were as follows:

- Use the pre-segmented choriocapillaris slab as the starting point for the review
- Manually adjust the width of the choriocapillaris slab to 40 µm
- Starting from the inner choroid gradually and manually move the entire slab along the axial direction from the inner choroid to the choriocapillaris, RPE, outer retina/avascular zone up to the inner plexiform layer. While scrolling through the depth resolved cross-sectional scan, the MNV, if present with active blood flow, or parts of it, as it is actually a three-dimensional structure, start to appear on the en face projection. At a specific depth, variable for each case, the network appears at its clearest and largest and then begins to fade as the slab continues to move towards the inner retina. Through this dynamic inspection, the grader determines the position of the slab where the neovascular complex is best visualised in terms of size and clarity. The grading is performed at the location where the neovascular complex is visible at its largest and clearest. Combining information from blood flow signals from both the enface and the cross-sectional flow signals at that location, is the optimal approach for estimating the anatomical localisation of the neovascular complex and so classify it into Types 1, 2 and 3.
- No segmentation errors were manually corrected, but their presence was recorded
- The same approach to OCTA interpretation was used for all the analysis
- Signal strength was good (20–25) as per standard parameters used in clinical imaging.

## Results

### Subject characteristics

Thirty-two eyes of 32 patients with treated stable nAMD were included in the study and underwent study imaging between September 2019 and March 2020. This study included 10 (31.3%) male and 22 (68.7%) female subjects. There were 20 (62.5%) left and 12 (37.5%) right eyes. Subjects had a mean (± SD) visual acuity of 69.7 ± 8.7 ETDRS letters and had undergone a mean (± SD) of 16.4 ± 8.7 intravitreal injection treatments at baseline. The imaging visit occurred at a mean number of 153.1 ± 85.5 weeks from baseline. No macular haemorrhage (subretinal, intraretinal or pre-retinal) was noted on slit-lamp biomicroscopy at this 12-week treatment visit.

### SD-OCT image analysis

Evaluation of SD-OCT images in the Moorfields Eye Hospital Reading Centre confirmed no significant image artefact preventing grading in any of the 32 SD-OCT macular scans. Evaluation confirmed that no SD-OCT images had signs of active nAMD (subretinal or intraretinal fluid). Other OCT features of AMD were noted including well defined subretinal hyperreflective material (13, 40.6%), pigment epithelial detachment (31, 96.9%), outer retinal tubulation (10, 31.2%) and macula atrophy (19, 59.4%).

### OCTA image analysis

Evaluation of OCTA images in the Moorfields Eye Hospital Reading Centre confirmed that 31/32 (96.9%) of OCA images were gradeable (1 OCTA image had significant signal attenuation). OCTA imaging artefacts were noted in 28/32 (87.5%) of OCTA images taken (all of which were projection artefacts).

The mean (± SD) OCTA lesion size was 3.6mm^2^ ± 4.6mm^2^. There was detectable MNV blood flow in 27 (87.1%) eyes. Analysis of MNV morphology confirmed MNV maturity in 29 (93.5%) of eyes, consisting of 10 non-specific, 10 tangled and 3 deadtree phenotypes. Of the immature MNV phenotype, there was 1 non-specific and 1 medusa subtypes.

**Fig. 1:**
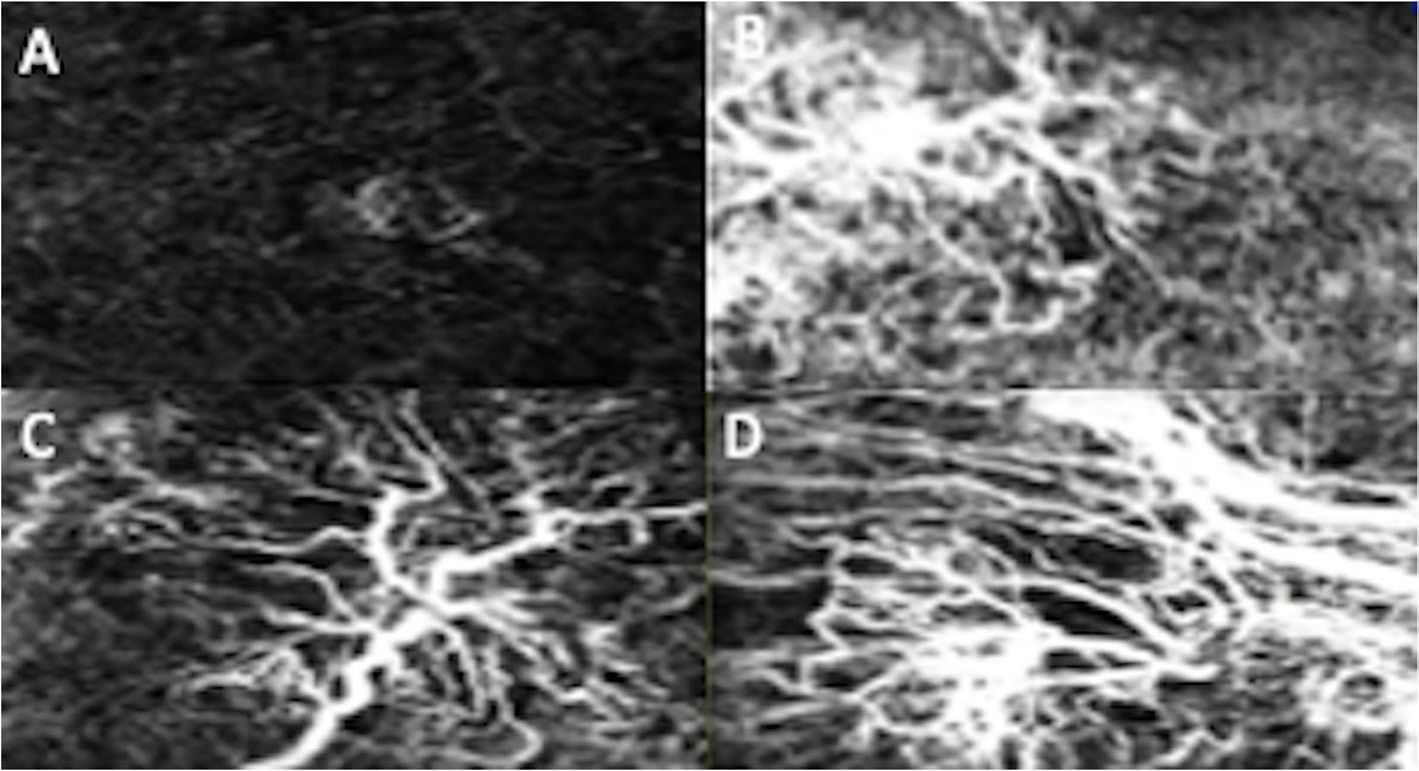
Optical coherence tomography angiography imaging showing examples of macular neovascularization phenotypes: non-specific (**A**), medusa (**B**), deadtree (**C**), tangled (**D**)

**Fig. 2:**
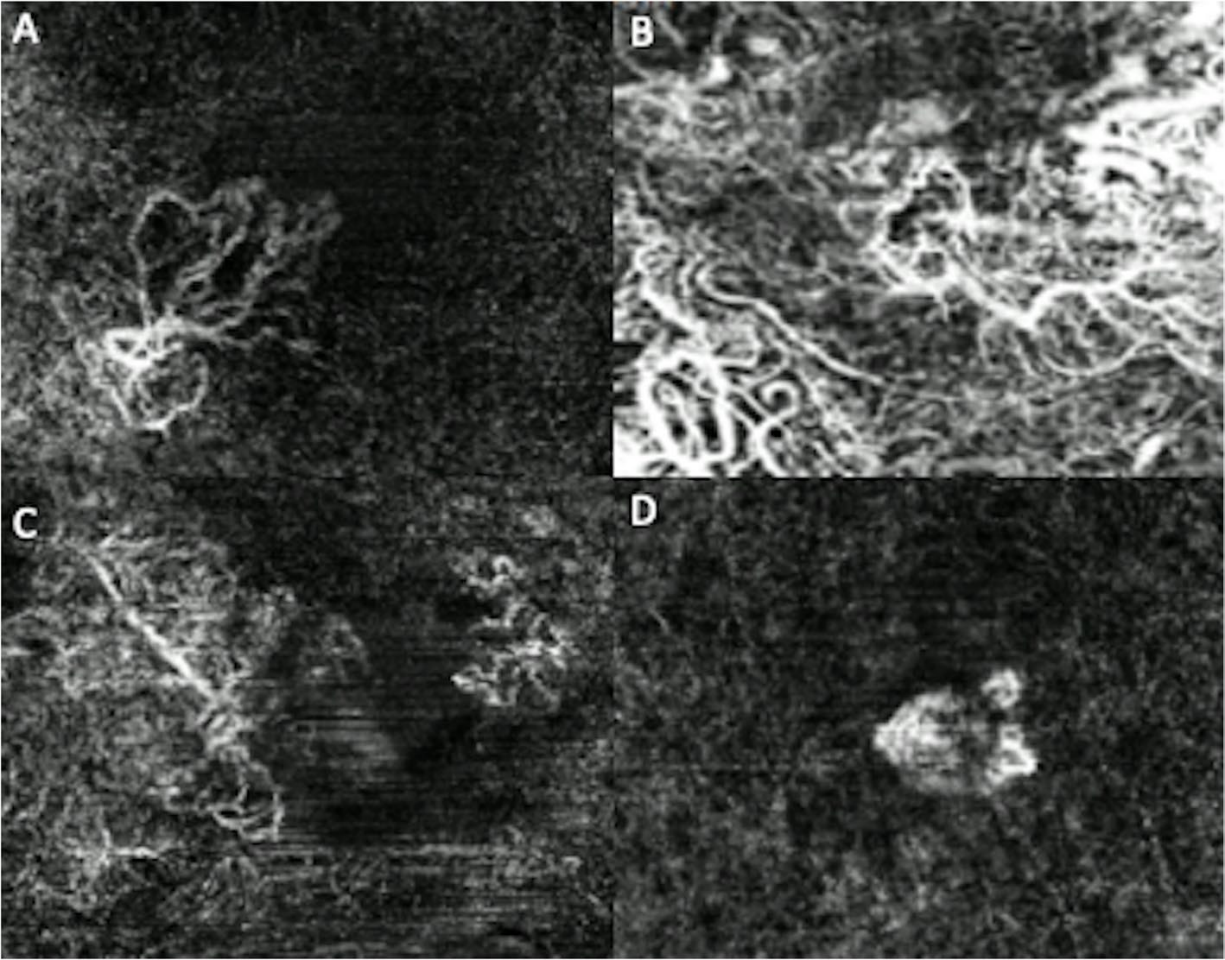
Optical coherence tomography angiography imaging showing examples of macular neovascularization features: central feeder vessel (**A**-**B**), hyporeflective halo (**C**), capillary fringe (**D**)

OCTA image analysis of qualitative features of disease activity included presence of MNV peripheral halo (29, 93.5%), capillary fringe (1, 3.2%) and central feeder vessel (19, 61.3%). Examples of OCTA morphologies and qualitiative features of disease activity in subjects in this study are shown in Figs. 1 and 2 respectively.

## Discussion

We evaluated OCTA derived features of MNV disease activity in patients with treated stable nAMD. This included presence of stable MNV phenotype (most commonly non-specific, tangled and deadtree morphology) as well as specific features of MNV activity include presence of a feeder vessel, surrounding hyporeflective halo and absence of capillary fringe. We suggest that these features are useful biomarkers to confirm MNV stability when used concurrently with conventional structural OCT assessment. They could be used to help guide treatment decisions and plan appropriate treatment intervals for patients with nAMD. Nearly all our OCTA images were of sufficient quality to allow grading by our specialist Reading Centre; projection artefact was present in the majority of cases but did not impede grading. Further evaluation of OCTA in nAMD is of course necessary but OCTA technology may allow for the ability to characterise the risk of disease reactivation particularly useful when planning long-term treatment strategies and clinical protocols.

Interestingly, OCTA visualised blood flow was still visible in the majority of these treated MNV. It would appear therefore that MNV consist of persistent active vessels despite no ongoing subretinal or intraretinal fluid on structural OCT examination. The clinical significance of OCTA visualised blood flow therefore is unclear; other studies have also found persistent MNV blood flow in eyes treated with anti-VEGF agents and which now appear clinically stable. [19] It would be interesting to investigate how the pattern of blood flow alters after treatment and with the development of disease activity. Other blood flow characteristics may provide further quantitative measurements useful for assessment of disease activity. [20] As previously described by Al-Sheikh et al, we also noted the presence of a MNV feeder vessel after treatment in these previously treated eyes. [13] The absence of a capillary fringe vessels in our study, akin to the alteration in branching of small vessels after treatment has also been identified in other studies. [14, 17, 21] It has been suggested that treatment with anti-VEGF agents results in an alteration in MNV morphology with the establishment of a dense feeder vessel (and a reduction in smaller peripheral vessels. [13, 17] Previous study has suggested that the prominent central feeder vessel develops over time and is less responsive to anti-VEGF agents. [13, 22, 23]. It is not possible to assess recurrence in this cross-sectional study. It would therefore be useful to investigate these long-term changes after reactivation and how they respond to re-treatment in a long-term cohort study. It is possible therefore that presence of a capillary fringe is an additional biomarker of disease activity in nAMD and can be evaluated, in addition to conventional structural OCT features, when considering stopping treatment in patients with nAMD. Future research should assess the significance of capillary fringe in influencing the continuing need for treatment beyond OCT structural features alone. The presence of capillary fringe could help to identify those eyes with high chance of relapse, despite no macular fluid.

Our study was limited in sample size, and further studies with more subjects would of course be useful. This study however was a prospective imaging study with a well-defined cohort of subjects. Imaging was performed by a clinical trials-certified technician and analysis of OCTA imaging was provided by the Moorfields Eye Hospital reading centre. Interestingly, SRHM appeared to be present in many cases, with concurrent signs of chronic and advanced nAMD (outer retinal tubulation and atrophy). This is arguably unusual and may reflect the sample size described; further evaluation in larger studies is required. Indeed, the presence of the SRHM, PED and retinal atrophy in a relatively significant proportion of cases could have affected analysis by inducing artefact in OCTA interpretation. Further study is of course indicated with assessment of MNV of different aetiology and utilising different forms of OCTA technology. Other putative quantitative features of disease activity including MNV vessel area and lacunarity can be included in future studies assessing disease activity. It would be important to determine the accuracy of manual OCTA segmentation techniques and the repeatability of OCTA derived lesion size in treated stable nAMD. We note that study images were assessed by a single grader potentially increasing grader error compared to double graded imaging studies. 3-Dimensional analysis of neovascular complexes may be useful in helping to investigate disease activity. [24]

## Conclusions

In summary, we describe OCTA findings of stability in treated nAMD including development of a mature MNV phenotype, presence of central feeder vessel, absence of capillary fringe and hyporeflective halo are possible biomarkers of stable disease activity in treated nAMD. We hope that further description of neovascular complexes with OCTA technology can help to inform our understanding of nAMD and be used to create new paradigms for treatment decisions with anti-VEGF agents.

## Supplementary Information

**Additional file 1: DANA Study OCT-A Grading Protocol (Moorfields Ophthalmic Reading Centre).** Study Reading Protocol for analysis of optical coherence tomography angiography (OCTA) imaging of eyes including detection of artefacts (projection, motion, segmentation), analysis of neovascularization blood flow and total lesion size, and description of morphology (maturity, phenotype and vascular branching).

## Data Availability

All data generated or analysed during this study are included in this published article.

## Abbreviations

AMD: Age related macular degeneration
Anti-VEGF: Anti-vascular endothelial growth factor
ETDRS: Early Treatment Diabetic Retinopathy Study
MNV: Macular Neovascularisation
nAMD: neovascular age related macular degeneration
OCT: Optical coherence tomography
OCTA: Optical coherence tomography angiography
PED: Pigment Epithelial detachment
SD: standard deviation
SD-OCT: spectral domain optical coherence tomography
