# Supplementary material for: Biomarkers of macular neovascularisation activity using optical coherence tomography angiography in treated stable neovascular age related macular degeneration
Source: BMC Ophthalmol. 2023 Feb 14;23:68. doi: 10.1186/s12886-022-02749-5 (PMC9926859; doi:10.1186/s12886-022-02749-5)

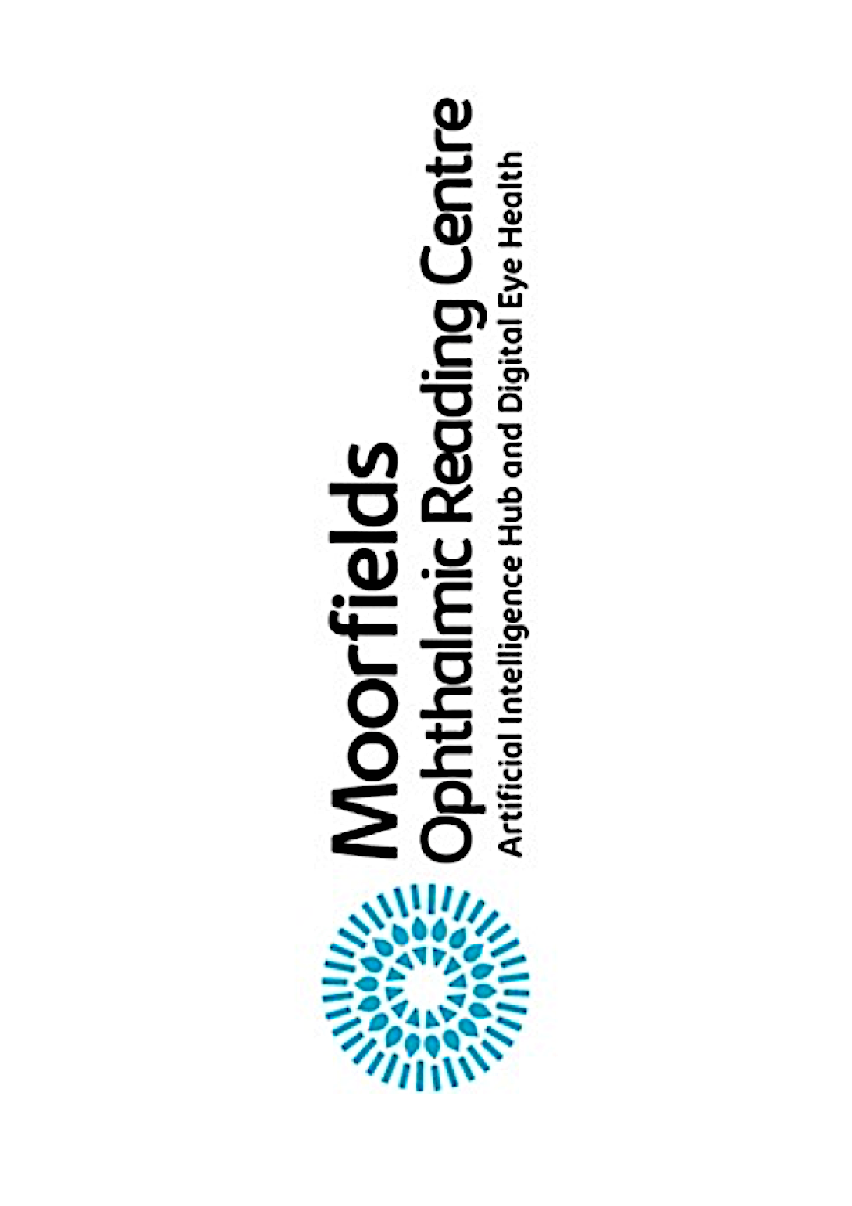


**DANA STUDY – OCT-A Grading Protocol**

**Moorfields Ophthalmic Reading Centre**

**Author: Konstantinos Balaskas**

**OCTA Gradable Image** (Gradable) – yes / no

**OCTA Image Artefact** yes /no

**If yes,** type of OCTA artefact:

**Projection**


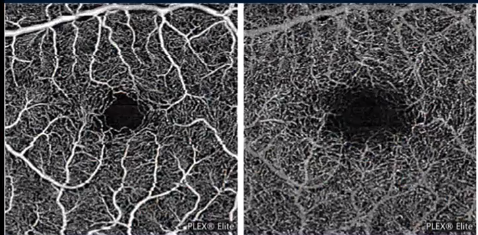


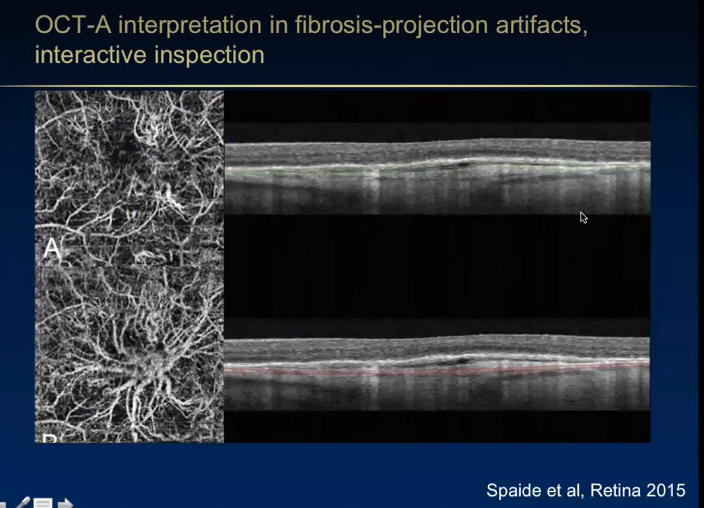


**Motion**


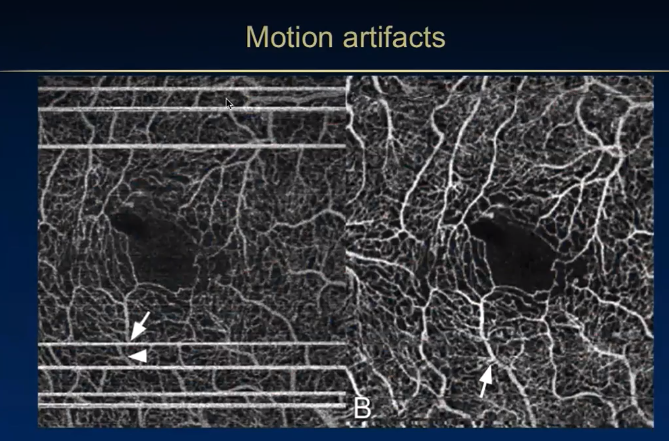


**Segmentation +/- signal attenuation**


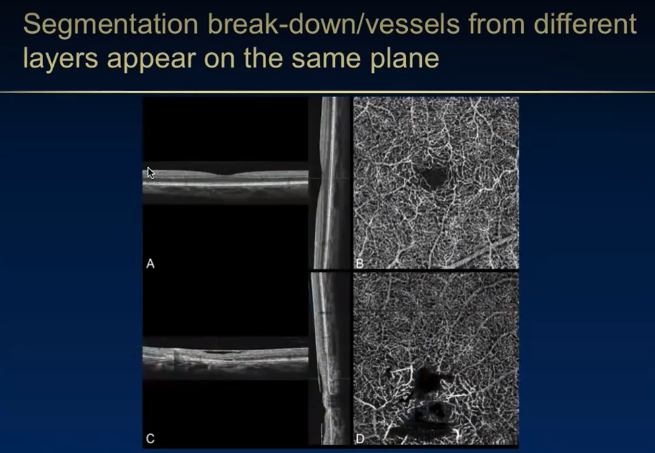


**OCTA High PED**:


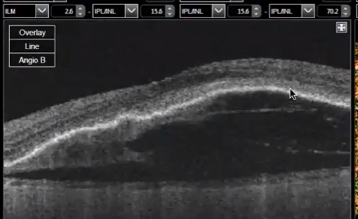


**OCTA CNV Blood Flow (detectable CNV):** yes/no (example of flow below)


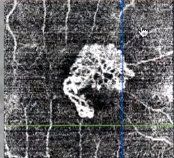


**OCTA CNV Size (mm2):** Application of the HEYEX software surface measurement tool

**OCTA CNV Type:** Type 1,2,3

1 under RPE


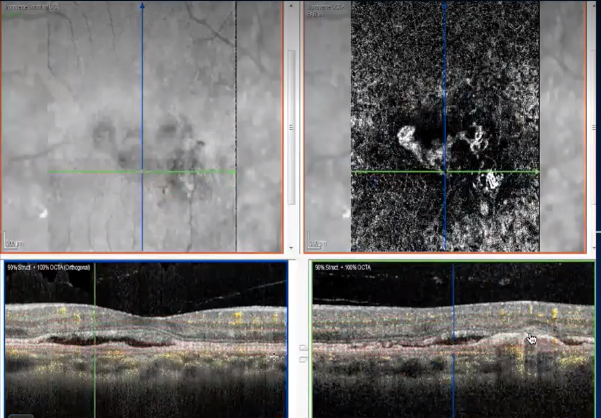


2 above RPE


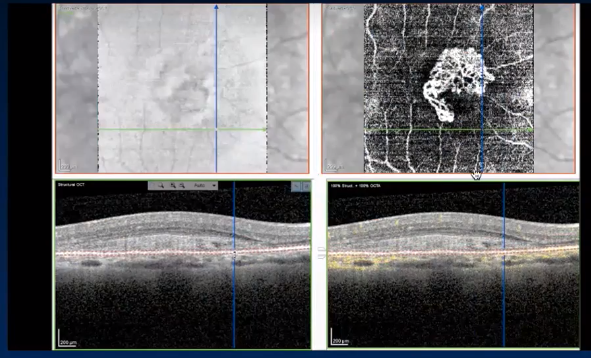


3 Retinal Angiomatous Proliferation


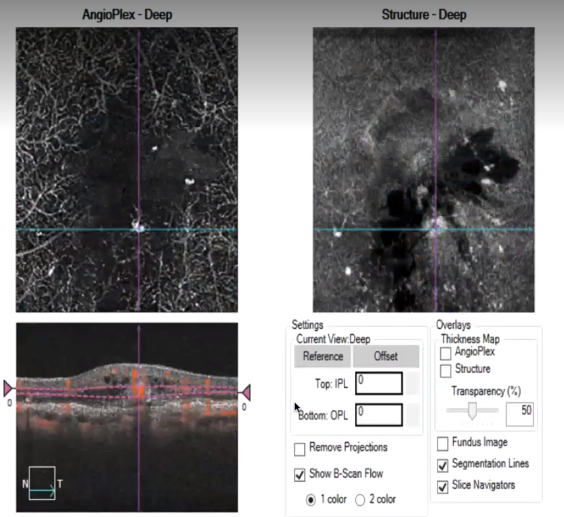


**OCTA CNV Maturity:** mature /immature

Mature: if the vascular network involved loosely packed and larger (trunk) vessels, with a small branching index and loss of the capillary fringe

Example:


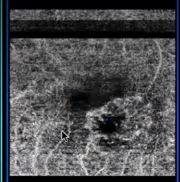


Immature: if they harbour a dense vascular network with a high branching index, high number of anastomoses and a smooth outline


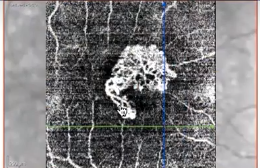


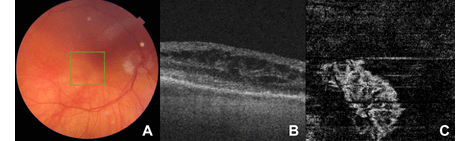


**OCTA CNV Mature** **Phenotype**: tangled / dead tree /nonspecific

Tangled

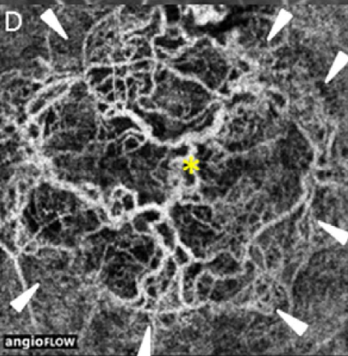

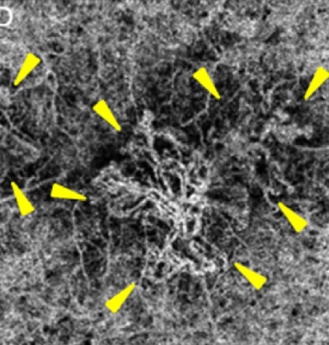


A Comparison Between Optical Coherence Tomography Angiography and Fluorescein Angiography for the Imaging of Type 1 Neovascularization – Tangled vessels

Dead tree:


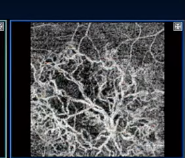


Non-specific


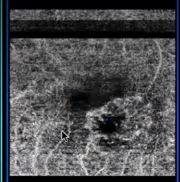


**OCTA CNV Immature** Phenotype: medusa-shaped/glomerulus shaped /nonspecific

Medusa shaped:


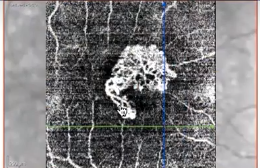


Glomerulus shaped


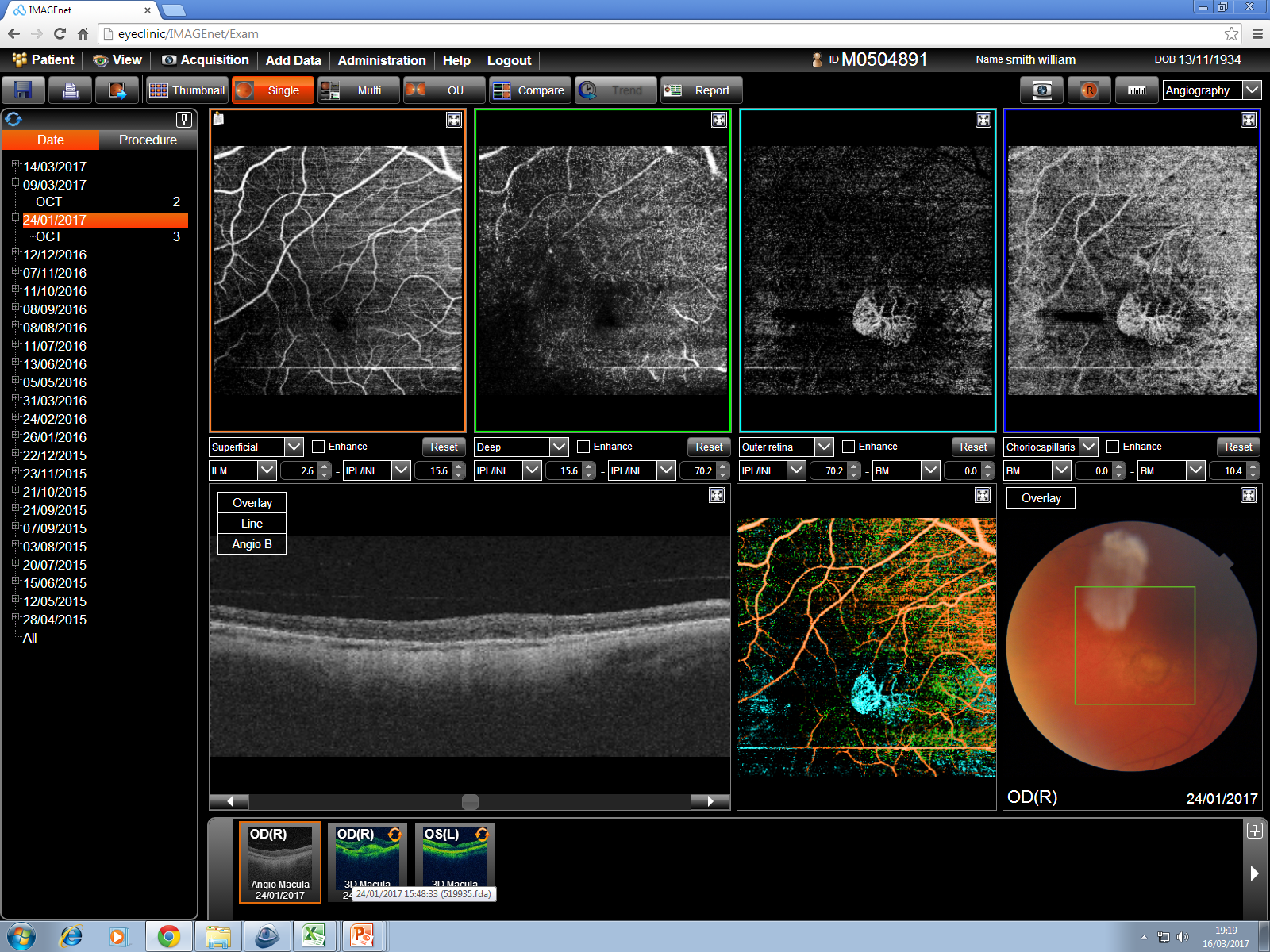


**OCTA Halo**: yes / no

Example: Yes


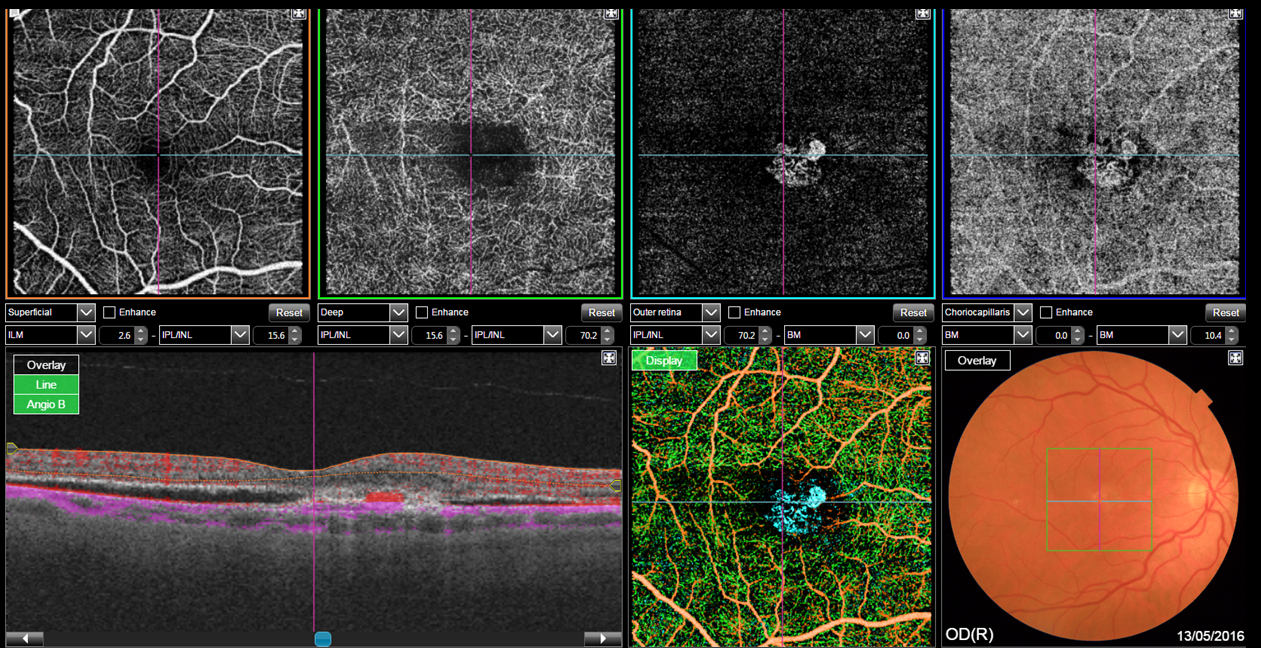


**OCTA Capillary Fringe**: all immature lesions will have this by definition. Smooth and dense outline of the network can be observed.


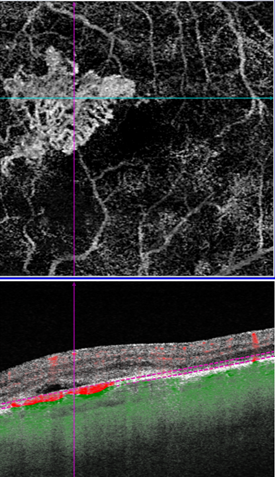


**OCTA Central Feeder Vessel**: when you can clearly see a central feeder vessel


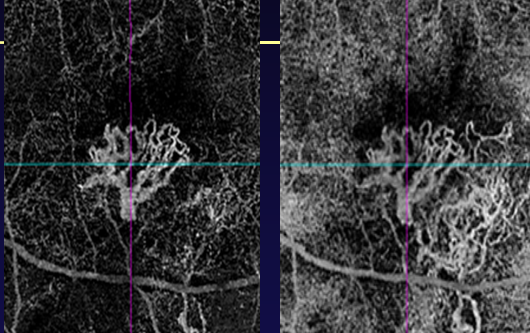

Supplement: Supplementary file 1 — Additional file 1: DANA Study OCT-A Grading Protocol (Moorfields Ophthalmic Reading Centre). Study Reading Protocol for analysis of optical coherence tomography angiography (OCTA) imaging of eyes including detection of artefacts (projection, motion, segmentation), analysis of neovascularization blood flow and total lesion size, and description of morphology (maturity, phenotype and vascular branching). [file 12886_2022_2749_MOESM1_ESM.docx]
